# Metabolic dependency of malonate protection against cardiac ischemia–reperfusion injury

**DOI:** 10.1007/s00395-026-01206-4

**Published:** 2026-08-17

**Authors:** Qian Wang, Xin Hu, Yang Xiao, Rianne Nederlof, Panagiota-Efstathia Nikolaou, Christina Chania, Diane Bakker, Amrita Kim Singh, Markus W. Hollmann, Nina C. Weber, Bauke Schomakers, Michel van Weeghel, Coert J. Zuurbier

**Affiliations:** 1 Laboratory of Experimental Intensive Care and Anesthesiology, Department of Anesthesiology, Amsterdam Cardiovascular Sciences, Amsterdam UMC, University of Amsterdam, Located AMC, Meibergdreef 9, 1105 AZ Amsterdam, The Netherlands; 2 Institute fur Herz- und Kreislaufphysiologie, Heinrich-Heine-Universitat, Dusseldorf, Germany; 3 Laboratory of Pharmacology, School of Pharmacy, National and Kapodistrian University of Athens, Athens, Greece; 4 Laboratory Genetic Metabolic Diseases, Location Academic Medical Center, Amsterdam University Medical Centers, University of Amsterdam, Amsterdam, The Netherlands; 5 Core Facility Metabolomics, Location Academic Medical Center, Amsterdam University Medical Centers, University of Amsterdam, Amsterdam, The Netherlands; 6 Amsterdam Gastroenterology Endocrinology and Metabolism Institute, Amsterdam, The Netherlands

**Keywords:** Malonate, Ischemia–reperfusion injury, pH, Insulin, Lactate, Glycogen, Metabolism

## Abstract

Malonate, an inhibitor of mitochondrial succinate dehydrogenase (SDH), protects against ischemia–reperfusion injury (IRI) when given at early reperfusion, making it a promising therapeutic agent for acute myocardial infarct (AMI) and stroke patients. Prior to clinical studies, it is essential to identify potential translational roadblocks. One such barrier is the metabolic signature of the heart. Here, we explored whether and how the metabolic signature of the heart drive cardiac IRI and modulate malonate protection. Langendorff-perfused mouse hearts were subjected to 30 min ischemia (I) followed by 90 min reperfusion (R), with or without 5mM disodium malonate administered during the first 5 min reperfusion. Cardiac IRI and malonate protection were examined under four metabolic conditions increasing in metabolic complexity: glucose-only (G), glucose + glutamine (GG), GG + fatty acid (F), F + insulin (Ins). Metabolomic profiling and survival kinases were evaluated at end-ischemia and at 7 min of reperfusion ± malonate. Additional experiments examined the effects of glucose, lactate, and low pH on malonate efficacy. IRI (% infarct size) increased with metabolic complexity: 32 ± 11% (G), 38 ± 11%(GG), 52 ± 14% (F), and 68 ± 10% (Ins). Malonate reduced IS% in the G (-27%), GG (-45%) and F (-23%) groups, but not in the Ins group (-12%, not significant). Malonate increased the glycolytic activator and protectant fructose-1/2,6-bisphosphate, mitochondrially bound hexokinase 1 and ATP across all metabolic conditions, confirming measurements showing cellular malonate uptake in all metabolic conditions. Loss of protection correlated with elevated pre-ischemic glycogen levels, end-ischemic lactate levels and delayed ischemic contracture, both indicative of prolonged glycolysis during ischemia due to glycogen loading, and increased glycolytic intermediates during early reperfusion hallmarks of cardiac glucose loading. Indeed, protection was restored in the insulin group when glucose was withheld prior to ischemia. With glucose present before ischemia, elevated lactate and lower pH were observed at onset reperfusion. Further experiments revealed that lower pH—not elevated lactate—at reperfusion was responsible for the loss of malonate protection. Disodium malonate (120 mg/kg) was also unable to reduce infarct size in an in vivo rat model of cardiac IRI employing similar insulin levels as used in the Langendorff-perfused mouse heart model. While malonate offers robust protection against cardiac IRI across various metabolic environments, its efficacy is compromised under conditions of high ischemic glycogen breakdown, which leads to severe acidosis at the onset of reperfusion that was associated with the loss of malonate protection.

**Supplementary Information:**

The online version contains supplementary material available at https://doi.org/10.1007/s00395-026-01206-4.

## Introduction

Insufficient energy supply and intracellular acidosis resulting from excessive anaerobic glycolysis during ischemia, followed by a burst of ROS and calcium during early reperfusion, are well-established mechanisms of acute cardiac ischemia–reperfusion injury (IRI) [17,18,42]. These IRI mechanisms, and protection thereof, are largely driven by specific alterations in the metabolism of the heart [31, 53]. Indeed, by profiling the metabolic signatures of ischemic organs, Chouchani et al. found that succinate was a major metabolite that increased during the ischemic episode and contributed to the burst of ROS upon reperfusion [5]. Upon reperfusion, approximately half of the accumulated succinate is quickly oxidized into fumarate by succinate dehydrogenase (SDH), favoring a reduced Coenzyme Q pool and thus creating a strong proton motive force that drives reverse electron transport (RET)-ROS production at Complex I [5, 30]. Therefore, preventing RET by inhibiting SDH has been proposed as a promising target to restrict early reperfusion ROS production and therefore IRI [30]. Malonate, a SDH inhibitor, was shown to protect the heart against both acute IRI, long-term post-ischemia heart failure and ischemic stroke, making it a promising protective intervention for AMI/stroke patients [1, 24, 29, 34, 39, 40].

Although numerous interventions and compounds have shown promise as cardioprotectants in preclinical studies, the translation from bench to bedside has proven to be unsatisfactory, likely due to the lack of rigorous and systematic preclinical evaluation [10, 23]. Myocardial infarction patients have a higher prevalence of metabolic syndrome, which is a cluster of diseases and cardiovascular risk factors associated with abnormal myocardial bioenergetics [51]. Moreover, preclinical studies have shown that different metabolic milieus can affect the efficacy of cardioprotective interventions, as exemplified by abolition of morphine preconditioning by glutamine, loss of insulin protection due to elevated fatty acids and loss of protection by ischemic preconditioning, NHE1 inhibition, and NAD^+^ precursor treatment by insulin [11, 12, 16, 38, 46]. The loss of cardioprotection in the presence of insulin has been ascribed to insulin-induced elevated glycogen breakdown during ischemia, activation of glycolysis, and/or activation of the RISK pathway [12, 38, 46]. Highly activated glycolysis and RISK pathway prevent further activation by cardioprotective interventions, resulting in loss of protection by these interventions [42, 50, 53].

It is unknown whether malonate protection is modulated by cardiac metabolism. We anticipated that malonate protection is affected by the metabolic milieu of the heart due to the predominantly metabolic nature of malonate protection. The effect of malonate on myocardial metabolites in glucose-only perfused mouse hearts during normoxia and at 5 min of reperfusion has been analyzed with ^1^H NMR spectroscopy [39]. Surprisingly, malonate only affected the metabolite succinate in these normoxic and reperfused glucose-only perfused hearts. In the current study, we aimed to conduct a more comprehensive evaluation of metabolic alterations induced by short-term malonate administration and the metabolic milieu by examining cardiac metabolomics in various metabolic milieus of the heart, at end-ischemia and at early reperfusion, with and without malonate treatment. Additionally, we examined the effects of malonate on well-known cardioprotective signaling pathways (RISK pathway, AMPK, mitochondrial-bound hexokinase (mtHK), oxidative stress) [42, 50] to determine whether protection by malonate is, at least partly, mediated through these signaling pathways beyond SDH inhibition.

Therefore, in this study, we addressed the following questions: (1) Is the metabolic milieu a determinant of cardiac IRI?, (2) Is malonate protection dependent on the metabolic milieu of the heart?, (3) Does malonate affect cardiac metabolomics at early reperfusion, and if so, do cardiac metabolomics, at least partly, correlate with the protective effect of malonate?, (4) Does malonate affect cardioprotective signaling, and if so, does this signaling, at least partly, associate with malonate’s protection?, (5) What are the metabolic factor(s) that can abolish malonate efficacy?

## Methods

### Animals

Animal experiments were approved by the Animal Ethical Committee of the University of Amsterdam and conducted in accordance with Directive 2010/63/EU of the European Parliament and with the “Guide for the Care and Use of Laboratory Animals”. Male C57BL/6NCrl mice (10–14 weeks old; body weight 25 ± 2 g) were obtained from Charles River Laboratories, Germany. The mice were acclimatized to their new environment for at least seven days under standard housing conditions at the Animal Research Institute AMC, with free access to food and water.

### Heart isolation and Langendorff preparation

Mice were anesthetized with pentobarbital (95 mg/kg, intraperitoneally) and injected with the anticoagulant heparin (15 U). After the loss of withdrawal reflexes to hind limb toe pinch [55], tracheotomy was performed, and mechanical ventilation was initiated (50% O₂, 50% N₂) [35]. Aorta was cannulated in situ and hearts excised resulting in euthanasia of the animals. The hearts were immediately perfused with Krebs–Henseleit buffer (118 mM NaCl, 0.5 mM EDTA, 4.7 mM KCl, 2.25 mM CaCl₂, 1.2 mM MgSO₄, 25 mM NaHCO₃, 1.2 mM KH₂PO₄) and the respective substrates, then installed in the constant-flow Langendorff setup as reported previously [44, 47]. Perfusate was continuously gassed with 95% O_2_ and 5% CO_2_, resulting in a stable pH = 7.4. After removing the surrounding tissue, an apex cannula was inserted to release Thebesian flow from the left ventricle, and a left ventricular balloon was installed. Following a 20-min stabilization period, balloon volume was adjusted to obtain a diastolic pressure between 3 and 7 mmHg, and initial perfusion pressure was set between 75 and 85 mmHg. Hearts were continuously submerged in 37°C perfusate, and cardiac function was monitored and recorded. Hearts with a low heart rate (< 280 beats/min), a low left ventricular systolic pressure (< 60 mmHg), and a high flow (> 4 ml/min) were excluded from the experiments. A total of 7 hearts were excluded (body weight 23 ± 2 g), mainly because of arrhythmia (1), rupture of aorta resulting in high flow (1), low heart rate (3), dead during surgery (1) and technical error (1).

### Experimental groups

#### Series I: Metabolic milieu effects on cardiac IRI and malonate protection

**Fig. 1:**
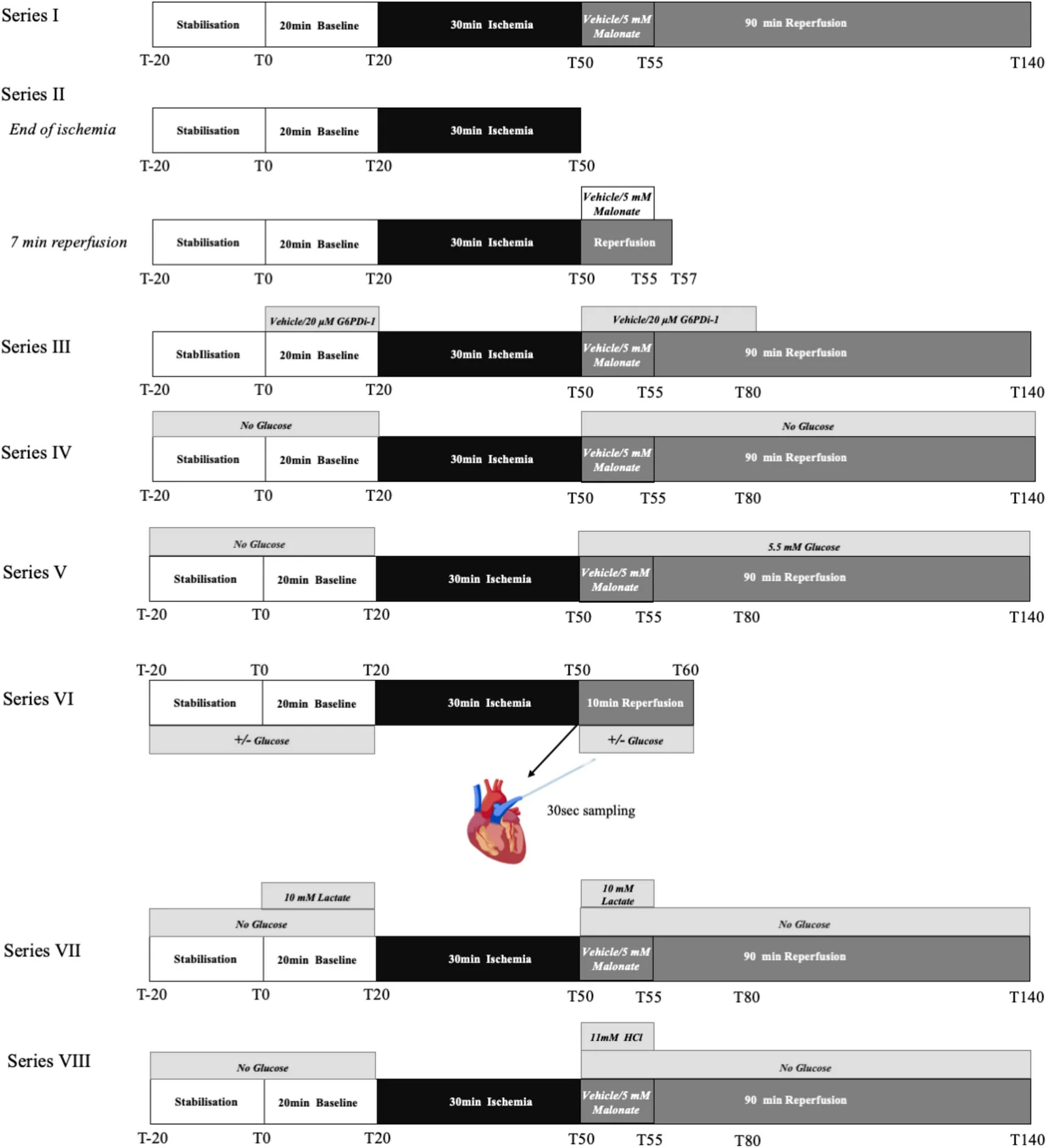
Experimental protocols for the different series of experiments. Series I, to determine effects of different metabolic milieus on cardiac ischemia–reperfusion injury (IRI) and protection by malonate (*n* = 8–19 biological replicates per group); Series II, to determine cardiac metabolomics and survival kinases as function of metabolic milieu (GG, F and Ins groups) and malonate at end-ischemia and early reperfusion (*n* = 7/8 biological replicates per group); Series III, examining malonate efficacy in the Ins group during the inhibition of the pentose phosphate pathway with G6PDi-1 (*n* = 6 biological replicates per group); Series IV, examining malonate efficacy for the Ins group with absence of glucose throughout the experiment (Ins-NG group) (n = 6 biological replicates per group); Series V, examining malonate efficacy in Ins group with absence of glucose before ischemia (*n* = 6 biological replicates per group); Series VI, sampling of venous effluent at onset reperfusion to determine lactate and pH for Ins group with and without glucose (*n* = 5/6 biological replicates per group); Series VII, examining malonate efficacy in the presence of 11 mM lactate in Ins group without glucose (*n* = 6/7 biological replicates per group); Series VIII, examining malonate efficacy in presence of low pH at onset of reperfusion for Ins group without glucose (*n* = 6 biological replicates per group)

In this series, the severity of cardiac IRI and cardioprotective efficacy of malonate were explored for four different metabolic perfusates. All isolated hearts, after 20 min stabilization and 20 min baseline, were subjected to 30 min ischemia and 90 min reperfusion (*n* = 8–19/group). Hearts were either treated with malonate (5 mM) or vehicle (saline) during the first 5 min of reperfusion. The perfusion and malonate treatment protocols are depicted in Fig. 1, series I. At the end of the experiments, the heart was stored at -20 °C for infarct size determination.

Four different metabolic groups were examined by adding metabolic substrates/hormones to the perfusate: G group: 11 mM glucose; GG group: 0.5 mM glutamine added to 11 mM glucose; F group: 0.2 mM palmitic acid bound to 1% albumin added to 5.5 mM glucose and 0.5 mM glutamine; Ins group: 30 mU/L insulin added to 5.5 mM glucose, 0.5 mM glutamine, and 0.2 mM palmitic acid bound to 1% albumin. Concentrations of insulin and metabolic substrates were chosen to reflect concentrations measured in the fed in vivo conditions of both mice and humans [21, 32].

#### Series II: Malonate and metabolic milieu effects on survival pathways and cardiac metabolomics

In this series, cardiac metabolites and protein levels of survival pathways were determined for three different metabolic groups (GG, F, and Ins) at end-ischemia (I), at 7 min reperfusion after ischemia (R) without malonate (control; Rco) and at 7 min reperfusion after ischemia with malonate treatment (malonate; Rma) during the first 5 min of reperfusion. Thus, a total of nine different experimental groups were examined (*n* = 7/8 per group). The notation of these groups is as follows: Ischemia group: *GG.I, F.I*, *Ins.I;* Reperfusion control group: *GG.Rco, F.Rco*, *Ins.Rco;* Reperfusion malonate group: *GG.Rma, F.Rma*, *Ins.Rma.*

Perfusion protocols are depicted in Fig. 1, series II. At the end of each protocol, fresh heart apex was used to obtain a mitochondrial fraction and the rest of the hearts was quickly frozen with liquid nitrogen and freeze-dried for metabolomics and western blotting. For mitochondria isolation, heart apex was homogenized (Sartorius, LabWrench, LabX, ON, Canada) at 1200 rpm (5 times × 5 s, on ice) in 1 ml homogenization lysis buffer (pH = 7.40) containing 0.25 M sucrose, 0.02 M HEPES, 1 mM dithiothreitol, 1 × HaltTM Protease & Phosphatase Inhibitor (Thermo Fisher Scientific, Waltham, MA – USA), 10 mM NaOH. Homogenates were immediately centrifuged at 10000g for 10 min at 4 °C. The precipitate (mitochondrial pellet) and the supernatant (cytosolic fraction) were collected, quickly frozen, and stored at -70 °C for further use.

#### Series III: Malonate protection and pentose phosphate pathway activity

To examine a possible role for pentose phosphate pathway (PPP) activation concerning the loss of malonate protection in the Ins group, two Ins groups (*n* = 6 per group) were examined in the presence of 20 µM G6PD-1, an inhibitor of the PPP. The concentration of the inhibitor was based on previous studies [9, 14, 43]. Perfusion protocol and infusion protocol are represented in Fig. 1, series III. G6PD inhibitor was given during 20 min baseline and 30 min reperfusion with or without malonate treatment.

#### Series IV: Malonate protection with absence of glucose throughout the whole experiment

To examine a possible role for glucose on effective malonate cardioprotection in the insulin group, glucose was removed from the Ins perfusate and malonate protection was evaluated (*n* = 6 per group). Glucose was removed from the Ins perfusate throughout the entire protocol of baseline, ischemia and reperfusion. In this no glucose group **(Ins-NG)**, preparation, perfusion protocol, and malonate treatment were performed as described in Fig. 1, series IV. For western blot protein detection at early reperfusion, the perfusion protocols are illustrated in Fig. 1, series II.

#### Series V: Malonate protection with absence of glucose prior to ischemia

To determine whether the loss of malonate protection in the Ins group was caused by the presence of glucose prior to ischemia, malonate protection was examined for hearts that were only perfused with glucose during reperfusion (Ins-RG, *n* = 6 per group). The perfusion protocol is depicted in Fig. 1, series V.

#### Series VI: Pre-ischemic glucose + insulin effects on ischemic contracture and venous lactate and pH at early reperfusion

To determine whether the pre-ischemic presence of glucose in the Ins perfusate affected lactate and proton (pH) levels released at the onset of reperfusion, the pulmonary artery venous outflow was sampled immediately at onset of reperfusion for the Ins group with (*n* = 5) and without glucose (*n* = 6). Lactate and pH in the venous effluent were determined by blood gas analysis (Siemens RapidPoint 500e), as shown in Fig. 1, series VI. In addition, time of ischemic contracture (TOC), as an indirect index of the amount of glycogen present in the heart at the start of ischemia [6, 38, 41], was determined in both groups.

#### Series VII: Malonate protection in the presence of high lactate at early reperfusion

To investigate whether the increased accumulation of end-ischemic lactate resulting in high venous lactate at early reperfusion with glucose + insulin perfusion was responsible for the loss of protection by malonate during reperfusion, hearts were perfused with 10 mM sodium L-lactate (Sigma-Aldrich, St. Louis, MO, USA) during the last 20 min before ischemia and the first 5 min of reperfusion. The hearts were perfused with the Ins perfusate without glucose throughout the whole experiment (**Ins-LNG,** *n* = 6/7 per group), with the protocol shown in Fig. 1, series VII.

#### Series VIII: Malonate protection in the presence of low pH at early reperfusion

Finally, to examine whether a low pH was responsible for the loss of malonate protection in conditions of glucose + insulin, hearts were perfused with 11 mM HCl without or with malonate (*n* = 6 for both groups) at the onset of reperfusion for 5 min. The concentration of HCl was selected based on previous studies [13, 49] and pilot studies in order to obtain a pH = 6.3 in the perfusate. Hearts were perfused with the Ins perfusate without glucose (Fig. 1, series VIII).

### TTC staining

At the end of 90 min reperfusion, the heart was weighed and then quickly frozen at -20 °C. For 2,3,5-triphenyltetrazolium chloride (TTC, Sigma-Aldrich, St. Louis, MO, USA) staining, the heart was cut into 5–6 slides of approximately 1 mm thickness and incubated in 1% TTC buffer (pH = 7.4) at 37 °C for 20 min on a shaker at 300 rpm. Then, heart slices were incubated in 4% formaldehyde for 2 h at room temperature in the dark and scanned. Images of the hearts were analyzed blinded for area at risk and infarct size (%IS, percentage of infarct size relative to area at risk) with SigmaScan Pro5 software.

### LC–MS

Metabolomics was performed as previously described, with minor adjustments [33, 52]. In a 2 mL tube, the following amounts of internal standard dissolved in water were added to each sample of ~ 3mg heart tissue: adenosine-^15^N_5_-monophosphate (5 nmol), adenosine-^15^N_5_-triphosphate (5 nmol), D_4_-alanine (0.5 nmol), D_7_-arginine (0.5 nmol), D_3_-aspartic acid (0.5 nmol), D_3_-carnitine (0.5 nmol), D_4_-citric acid (0.5 nmol), ^13^C_1_-citrulline (0.5 nmol), ^13^C_6_-fructose-1,6-diphosphate (1 nmol), ^13^C_2_-glycine (5 nmol), guanosine-^15^N_5_-monophosphate (5 nmol), guanosine-^15^N_5_-triphosphate (5 nmol), ^13^C_6_-glucose (10 nmol), ^13^C_6_-glucose-6-phosphate (1 nmol), D_3_-glutamic acid (0.5 nmol), D_5_-glutamine (0.5 nmol), D_5_-glutathione (1 nmol), ^13^C_6_-isoleucine (0.5 nmol), D_3_-lactic acid (1 nmol), D_3_-leucine (0.5 nmol), D_4_-lysine (0.5 nmol), D_3_-methionine (0.5 nmol), D_6_-ornithine (0.5 nmol), D_5_-phenylalanine (0.5 nmol), D_7_-proline (0.5 nmol), ^13^C_3_-pyruvate (0.5 nmol), D_3_-serine (0.5 nmol), D_6_-succinic acid (0.5 nmol), D_4_-thymine (1 nmol), D_5_-tryptophan (0.5 nmol), D_4_-tyrosine (0.5 nmol), and D_8_-valine (0.5 nmol). Subsequently, solvents were added to achieve a total volume of 500 µL methanol and 500 µL water. A 5 mm stainless steel bead was added and a Qiagen TissueLyser II was used for 5 min at 30 times/s to homogenize each sample, before the addition of 1 mL chloroform. After thorough mixing, samples were centrifuged for 10 min at 14.000 rpm. The polar top layer was transferred to a new 1.5 mL tube and dried using a vacuum concentrator at 60°C. Dried samples were reconstituted in 100 µL 6:4 (v/v) methanol:water. Metabolites were analyzed using a Waters Acquity ultra-high performance liquid chromatography system coupled to a Bruker Impact II™ Ultra-High Resolution Qq-Time-Of-Flight mass spectrometer. Samples were kept at 12°C during analysis and 5 µL of each sample was injected. Chromatographic separation was achieved using a Merck Millipore SeQuant ZIC-cHILIC column (PEEK 100 × 2.1 mm, 3 µm particle size). Column temperature was held at 30°C. Mobile phase consisted of (A) 1:9 (v/v) acetonitrile:water and (B) 9:1 (v/v) acetonitrile:water, both containing 5 mmol/L ammonium acetate. Using a flow rate of 0.25 mL/min, the LC gradient consisted of: Dwell at 100% Solvent B, 0–2 min; Ramp to 54% Solvent B at 13.5 min; Ramp to 0% Solvent B at 13.51 min; Dwell at 0% Solvent B, 13.51–19 min; Ramp to 100% B at 19.01 min; Dwell at 100% Solvent B, 19.01–19.5 min. Column was equilibrated by increasing flow rate to 0.4 mL/min at 100% B for 19.5–21 min. MS data were acquired using negative and positive ionization in full scan mode over the range of m/z 50–1200. Data were analyzed using Bruker TASQ software version 2021.1.2.452. All reported metabolite intensities were normalized to the sum of all adenosine nucleotides, as well as to internal standards with comparable retention times and response in the MS. Metabolite identification has been based on a combination of accurate mass, (relative) retention times, compared to the analysis of a library of standards.

### Western blotting

The mitochondrial fraction was resuspended with 1 ml lysis buffer containing 0.5% Triton X-100 and 10 µl of 100 mM glucose-6-phosphate to promote the release of hexokinase from mitochondria [54]. After 15 min of incubation at room temperature, the mitochondria-enriched homogenates were sonicated for 2 × 5 s and centrifuged for 1 min at 10,000 g, 4 °C. The supernatant was aliquoted and stored at -70 °C for Lowry assay and mitochondrial protein analysis by western blot techniques. For whole-heart analysis, 2 mg of freeze-dried heart tissue from each sample was homogenized on ice in 0.5 ml ice-cold homogenization medium (0.25 M sucrose, 0.02 M HEPES, and 1 mM dithiothreitol, 1 × HaltTM Protease & Phosphatase Inhibitor). The homogenates were supplemented with 0.5% Triton X-100 on ice for 10 min, after which samples were sonicated for 5 s and centrifuged for 2 min (10,000 g, 4 °C). The supernatant was aliquoted and stored at -70 °C for further measurement. Protein concentration was determined by the Lowry assay, after which the protein concentration of each sample was adjusted to a final concentration of 3 mg/ml. Next, samples were heated for 5 min at 95 °C, diluted 1:5 with 5 × sample buffer containing sodium dodecyl sulfate (SDS) and mercaptoethanol. The samples were aliquoted and stored at -70 °C.

For western blotting, 4–12% Bis–Tris precast SDS–polyacrylamide gel (Criterion TM XT, Bio-Rad, Criterion cell tank, Bio-Rad, Hercules, CA, USA), MOPS running buffer, and PVDF FL membrane (Immobilon®-FL PVDF, IPFL00010—Millipore) were used. 1 µl of the Precision Plus Protein™ All Blue Prestained Protein Standards (#1,610,373, Bio-Rad) and 10–15 µl of sample containing 3 mg/ml of protein were loaded onto the gels. The gels were then set to electrophoresis at 120 V for approximately 1.5 h using the Bio-Rad Powerpac. After electrophoresis, protein transfer from the gel to the PVDF membrane took place using 1× transfer buffer at 100 V for 30 min. Next, the membranes were blocked in Intercept® (TBS) blocking buffer (LI-COR, 927–60,001) for 1 h at a room temperature on a shaker. Finally, the membranes were incubated with the primary antibody diluted in Intercept® T20 (TBS) Antibody Diluent (LI-COR, 927–65,001) on an orbital shaker overnight at 4 °C. Primary antibodies were listed as follows: GAPDH, #ab174646, Abcam, 1:1000; VDAC, #ab15895, Abcam, 1:1000; #13141S, CST, 1:1000; 4-HNE, #ab46545, Abcam, 1:1000; HKI, #2024S, CST, 1:1000; HKII, #2867S, CST, 1:1000; Phospho-mTOR, #5536s, CST, 1:500; mTOR, #4517s, CST, 1:1000; Phospho-Akt (Ser473), #9271s, CST, 1:500; Akt, #2920s, CST, 1:1000; Phospho-AMPKa (Thr172), #2535S, CST; AMPKa, #2593S, CST. The membranes were then washed with ice-cold 0.1% TBST washing buffer three times for 5 min each on a shaker. Secondary antibodies (IRDye® 680RD Goat-anti-Rabbit IgG secondary antibody, LI-COR BioSciences 926–68,071; IRDye® 680RD Goat-anti-Mouse IgG secondary antibody, LI-COR BioSciences 926–68,070; or Goat-anti-Rabbit IgG secondary antibody, LI-COR BioSciences 926–32,211, all at 1:5000) were incubated for 1 h at room temperature, avoiding direct light, on an orbital shaker. After incubation, the membranes were washed thoroughly three times for 5 min each with 0.1% TBST washing buffer and then scanned with the Odyssey® CLx Imager software (LI-COR BioSciences version 5.1, Westburg).

### In vivo regional cardiac ischemia–reperfusion rat model

We employed a previously reported in vivo regional I/R model in the adult male Wistar rat [45]. Following instrumentation and surgery, all rats were left for 15 min stabilization before start of the experimental protocol. Then, the heart was subjected to 25 min of ischemia (LAD coronary artery ligation) followed by 120 min of reperfusion (ligation release). Successful myocardial infarction was confirmed by observation of pallor of the heart and apparent ST segment elevation on the ECG. Malonate (120 mg/kg; *n* = 4) or vehicle (saline; *n* = 4) was administered for 10 min through the cannulated vena jugularis at 0.3 ml/min/kg, starting at 5 min before reperfusion until 5 min of reperfusion. Area at risk and % infarct size were determined blindly as previously reported [45].

### In vivo plasma insulin levels

To obtain an estimate of plasma insulin levels in vivo, we sampled venous blood from the non-fasted in vivo rat model of I/R at 11 AM in the morning, just before start ischemia (*n* = 8). Blood was centrifuged and plasma stored at −80 ℃ until analysis by an ultrasensitive rat insulin ELISA (Mercodia).

### Cardiac glycogen levels

Hearts of C57BL/6N male mice (age 11–14 weeks) were isolated and perfused as reported above for the F (*n* = 4) or the Ins group (*n* = 4). Following 20 min stabilization and 20 min baseline perfusion, hearts were quickly frozen in liquid nitrogen and freeze-dried overnight. Cardiac glycogen levels were measured using a Mouse Glycogen ELISA Kit (MBS733396, My BioSource), according to manufacturers’ instructions. Glycogen content was normalized to heart dry weight.

#### Succinate in effluent isolated hearts

Hearts of C57BL/6N male mice (age 11–12 weeks) were isolated and perfused as reported above for the F group (*n* = 6) or the Ins group (*n* = 6). Following 20 min stabilization and 20 min baseline perfusion, hearts were subjected to 30 min ischemia. At the start of reperfusion, the first 2.5 ml of the effluent from hearts was collected and quickly frozen in liquid nitrogen and stored at -80 ℃. Succinate was quantified using gas chromatography–flame oxidation detection (GC-FID) method adapted from routinely used organic-acid analysis procedure. Succinate was identified with an Agilent 780B gas chromatograph based on its relative retention time and quantified using the internal-standard method (2-phenylbutyric acid and 4-phenylbutyric acid) and the experimentally established response factor for succinate.

#### Statistics

Results are presented as mean ± standard deviation (SD). The normal distribution was tested using Shapiro–Wilk test. Statistical analyses were conducted using an unpaired Student's t-test for comparisons between two groups, and one-way analysis of variance (ANOVA) followed by Holm–Šidák post hoc test for comparisons between more than two groups when data were normally distributed. For non-parametric results, Kruskal–Wallis test was used to detect significance, followed by Dunn’s test. A p value < 0.05 was considered statistically significant. All statistical analyses were performed using GraphPad Prism (GraphPad Software Inc., La Jolla, CA, USA).

## Results

### Metabolic milieu of the heart is a major determinant of cardiac IRI

**Fig. 2:**
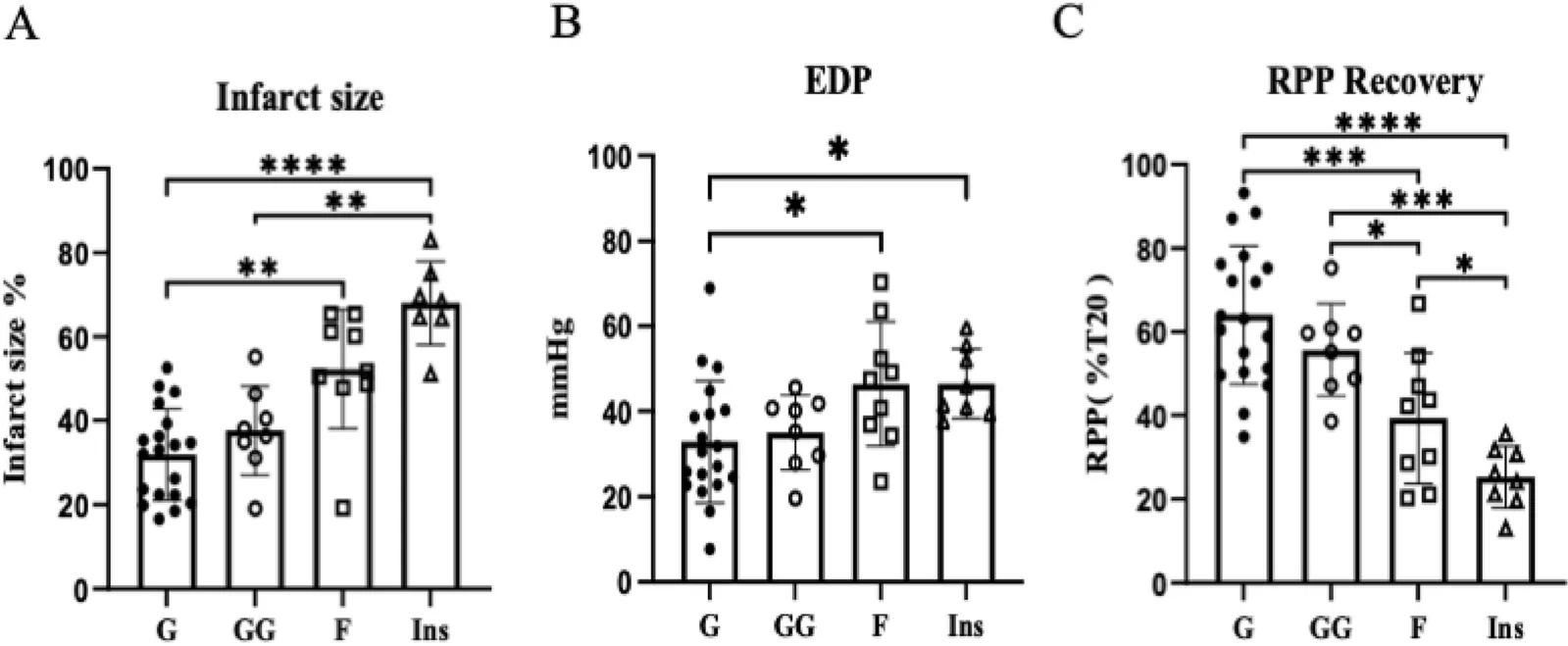
Cardiac ischemia–reperfusion injury (IRI) for the four metabolic conditions. Hearts were subjected to 30 min ischemia followed by 90 min reperfusion. **A** Infarct size (% of ventricular area) for the G (*n* = 19 hearts), GG (*n* = 8 hearts), F (*n* = 9 hearts) and Ins (*n* = 8 hearts) group. **B** End-diastolic pressure (EDP) after 90 min reperfusion as function of metabolic condition; **C** Recovery of Rate Pressure Product (RPP) for the different metabolic groups. RPP was calculated as (RPP_T140_—RPP_T20_)/RPP_T20_ × 100%, whereby T20 equals 20 min baseline perfusion. Kruskal–Wallis with Dunn’s post hoc test was used for comparison between all groups in panels A and B. One-way ANOVA with Holm–Šídák post hoc test was performed for comparison within groups in panels C

We first examined the effects of the different metabolic perfusates on the cardiac sensitivity to ischemia–reperfusion (Fig. 2A–C). Cardiac function at baseline showed no significant differences between the glucose-only and the other metabolic groups for most variables, except for a lower coronary flow with the addition of albumin/palmitic acid and a lower heart rate in the Ins group. However, the Rate Pressure Product (RPP) as an indirect indicator of energy turnover of the isolated heart was similar for all metabolic groups at baseline (Supplementary Table 1). The addition of glutamine was without any effect on cardiac IRI. In contrast, the addition of a low concentration of fatty acids to the cardiac perfusate increased cardiac IRI as reflected by increases in all cardiac IRI outcome variables as compared to the glucose-only group. Subsequently, adding insulin to the fatty acid perfusate numerically increased infarct size further and significantly decreased the recovery of cardiac function. Thus, the metabolic signature of the perfusate is a major determinant of the heart’s sensitivity to IRI, and the most-commonly used glucose-only perfusate in preclinical studies resulted in that hearts were most protected against IRI, whereas the most physiological metabolic composition of the perfusate resulted in the highest IRI sensitivity of the isolated mouse heart.

### Malonate protection is lost with insulin present

**Fig. 3:**
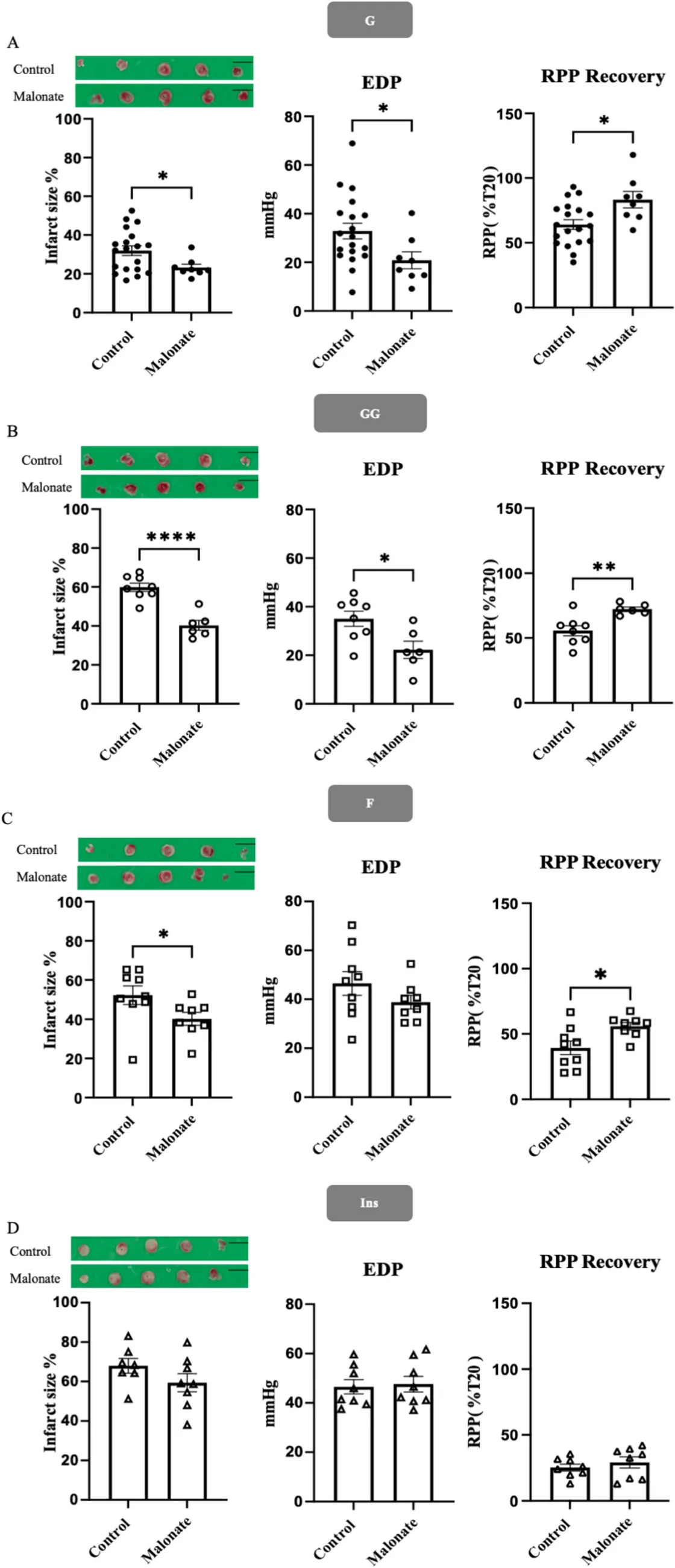
Cardioprotective efficacy of malonate for the four metabolic conditions. Hearts were subjected to 30 min ischemia followed by 90 min reperfusion. Saline (Control) or 5 mM malonate (Malonate) was administered during the first 5½ min of reperfusion. Infarct size (% of ventricular area, scale bar, 1 cm), end-diastolic pressure (EDP) at 90 min reperfusion, and recovery of Rate Pressure Product (RPP) were determined for the G **(A** *n* = 19 hearts in control, *n* = 8 hearts in malonate**)**, GG **(B** *n* = 8 hearts in control, *n* = 6 hearts in malonate**)**, F **(C** *n* = 9 hearts in control, *n* = 8 hearts in malonate**)** and Ins **(D** *n* = 8 hearts in control, *n* = 8 hearts in malonate**)** condition. RPP calculated as (RPP_T140_—RPP_T20_)/RPP_T20_ × 100%. A T-test (A, B, C[EDP, RPP Recovery] and D) or Mann–Whitney test (C[Infarct size]) was performed to compare the control and malonate groups

Next, we determined to what extent the metabolic milieu determined malonate’s potential to reduce cardiac IRI. Compared with each metabolic control group, malonate significantly decreased %IS by 27%, 45%, and 23% in the G, GG, and F groups, respectively (Fig. 3A, B, C). Similarly, recovery of cardiac function at 90 min reperfusion was improved by malonate in the G, GG, and F groups, whereas malonate also decreased end-diastolic pressure or contracture development for the G and GG groups (Fig. 3A, B, C). In contrast, malonate was unable to improve any of the three IRI outcome variables for the Ins group (Fig. 3D). Thus, adding insulin to a perfusate containing glucose, glutamine, and fatty acid abolished malonate’s protection that was present in perfusates without insulin. Subsequent analysis of cellular signaling and metabolomics for specific metabolic groups was performed trying to elucidate why malonate’s protection was lost in the presence of insulin.

### Malonate efficacy and plasma insulin levels in an in vivo rat model of regional cardiac I/R

To examine whether the ex vivo malonate effects were translated to the in vivo condition, we employed an established in vivo regional cardiac ischemia–reperfusion model in the rat. Plasma insulin levels in this model (15.3 ± 5 mU/L; Suppl. Figure 2A) were in a similar range as the 30 mU/L used for the isolated heart studies. Following 25 min ischemia and 2 h of reperfusion, area at risk (AAR) and infarct size (%IS) were 28 ± 9% and 55 ± 10%, respectively. Treating the animals with 120 mg/kg malonate during late ischemia and early reperfusion was without effect on infarct size (Suppl. Figure 2B), thereby supporting our results obtained in the isolated hearts.

### Malonate, cellular stress signaling, and oxidative stress

We first examined whether the loss of malonate protection could be explained by changes in cardioprotective stress signaling or oxidative stress. Because protection was lost when going from the F to the Ins metabolic condition, the focus is on the comparison between these two conditions. As anticipated, the cardioprotective RISK pathway (Akt and GSK-3β) was activated in the Ins group in contrast to the F group (Suppl. Figure 1A and 1G). However, opposite to the loss of protection by malonate in the Ins group, malonate at reperfusion was able to further activate the RISK pathway components Akt and GSK3β (but not mTOR) in the Ins group, and not in the F group. Thus, RISK pathway activation seems to play a compensatory role in conditions of abolished malonate protection and does not mediate malonate protection. In addition, although malonate resulted in decreased p-AMPK signaling in the Ins group, this malonate effect on AMPK was also observed for the F group (Suppl. Figure 1B and 1G). Clearly, malonate protection is also not mediated through AMPK activation. Increases in mitochondrial-bound hexokinase (mtHK) are known to protect against cardiac IRI [15, 28, 36]. Malonate protection in the F group was indeed associated with increased mtHK1. However, a similar increase in mtHK1 with malonate occurred in the Ins group (Suppl. Figure 1C and 1G). No changes in mtHK2 with malonate were observed for the F group, whereas mtHK2 was actually elevated with malonate in the Ins group (Suppl. Figure 1D and 1G). Oxidative stress was monitored by the ROS-induced lipid peroxidation product 4-HNE. We first confirm a burst of ROS at early reperfusion because 4-HNE was increased at reperfusion for both metabolic groups (Suppl. Figure 1E and 1H). However, we were unable to confirm that malonate reduced 4-HNE in the F group. Interestingly, malonate treatment in the Ins group was actually associated with increased 4-HNE (Suppl. Figure 1E and 1F). These data suggest that in our model, malonate protection in the F group is not associated with RISK/AMPK pathway activation, increased mtHK2 levels, or reduced 4-HNE levels. Most importantly, the loss of malonate protection in the Ins group cannot be explained by impaired RISK/AMPK activation or decreased mtHK levels as compared to malonate treatment in the F group, but possibly by malonate-induced ROS production during reperfusion.

### Cardiac metabolomics during ischemia and reperfusion

**Fig. 4:**
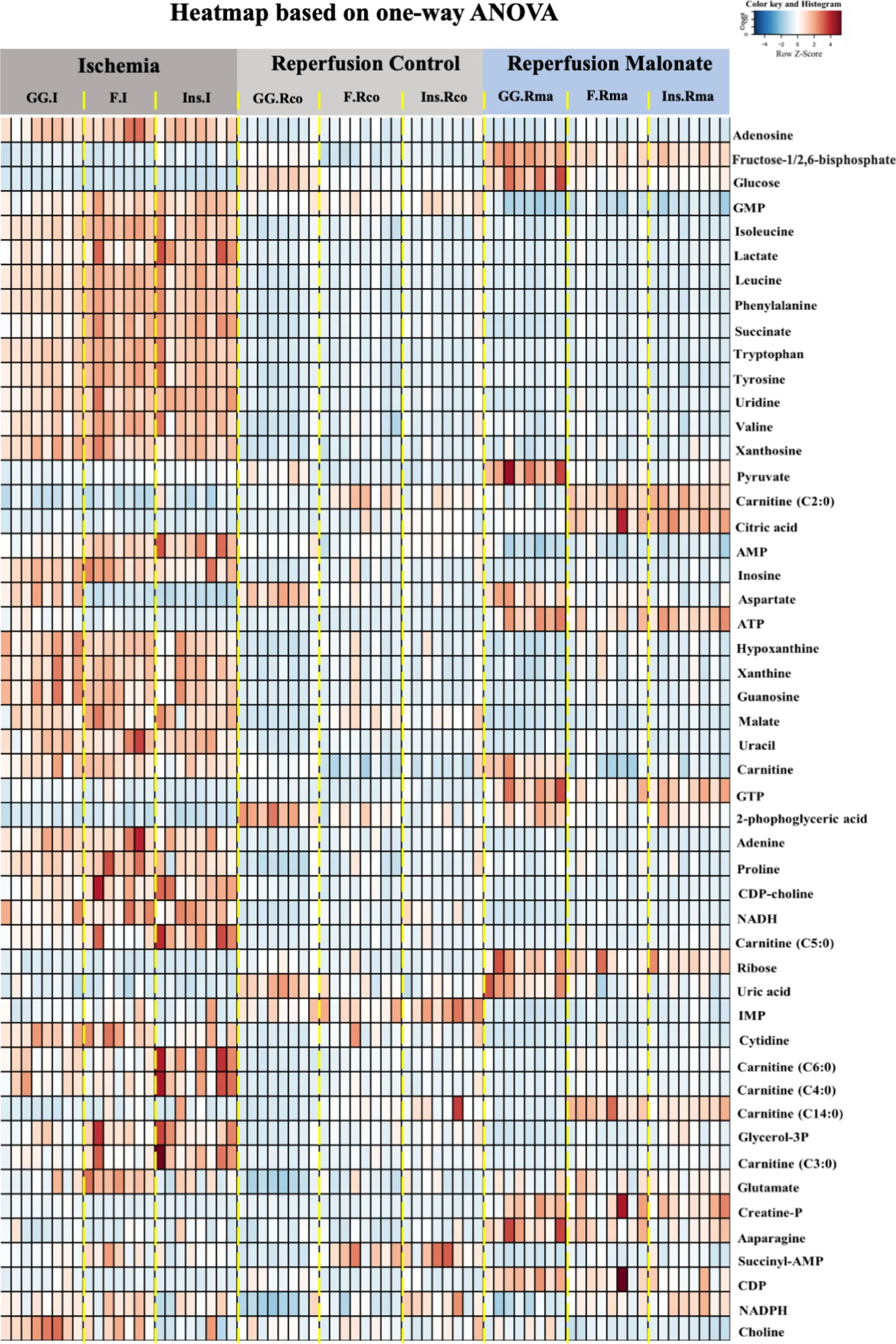
Heat map of cardiac metabolic intermediates. Metabolic intermediates are shown at end-ischemia and 7-min reperfusion with or without malonate treatment for the GG, F and Ins metabolic conditions. *N* = 7 hearts for F.Ins group, *n* = 8 hearts for all other groups. The top 50 metabolites are shown according to increasing p value of a one-way ANOVA

We first determined whether malonate uptake by the heart at 7 min reperfusion was affected by the different metabolic perfusates. Malonic acid, normalized to the internal standard citrate and dried heart weight, was equally increased with malonate administration for all three GG, F, and Ins groups relative to no malonate treatment (Suppl. Figure 2C). Thus, differences in malonate protection with different metabolic perfusates cannot be explained by changes in malonate uptake. Cardiac metabolomics for GG, F, and Ins groups at end of ischemia and at early reperfusion without and with malonate are presented (Fig. 4). For numerous cardiac metabolites, the end-ischemic period separated clearly from the controlled reperfusion period, which in its turn separated clearly from the malonate reperfusion, independent of the three metabolic conditions (Fig. 4). For all three metabolic groups, besides the expected ischemic accumulation of succinate and lactate, various amino acids and purines (adenine nucleotide degradation products) also accumulated at end-ischemia, which were all quickly reduced upon reperfusion. Malonate reperfusion versus control reperfusion resulted in increases in fructose-1/2,6-bisphosphate (FBP), ATP, GTP, ribose, and creatine phosphate for all three metabolic groups, suggesting activated glycolysis and improved cardiac energetics at early reperfusion with malonate (Fig. 4). However, no metabolite at early reperfusion became apparent that clearly characterized malonate treatment in the Ins group from that of malonate treatment in the F or GG group.

**Fig. 5:**
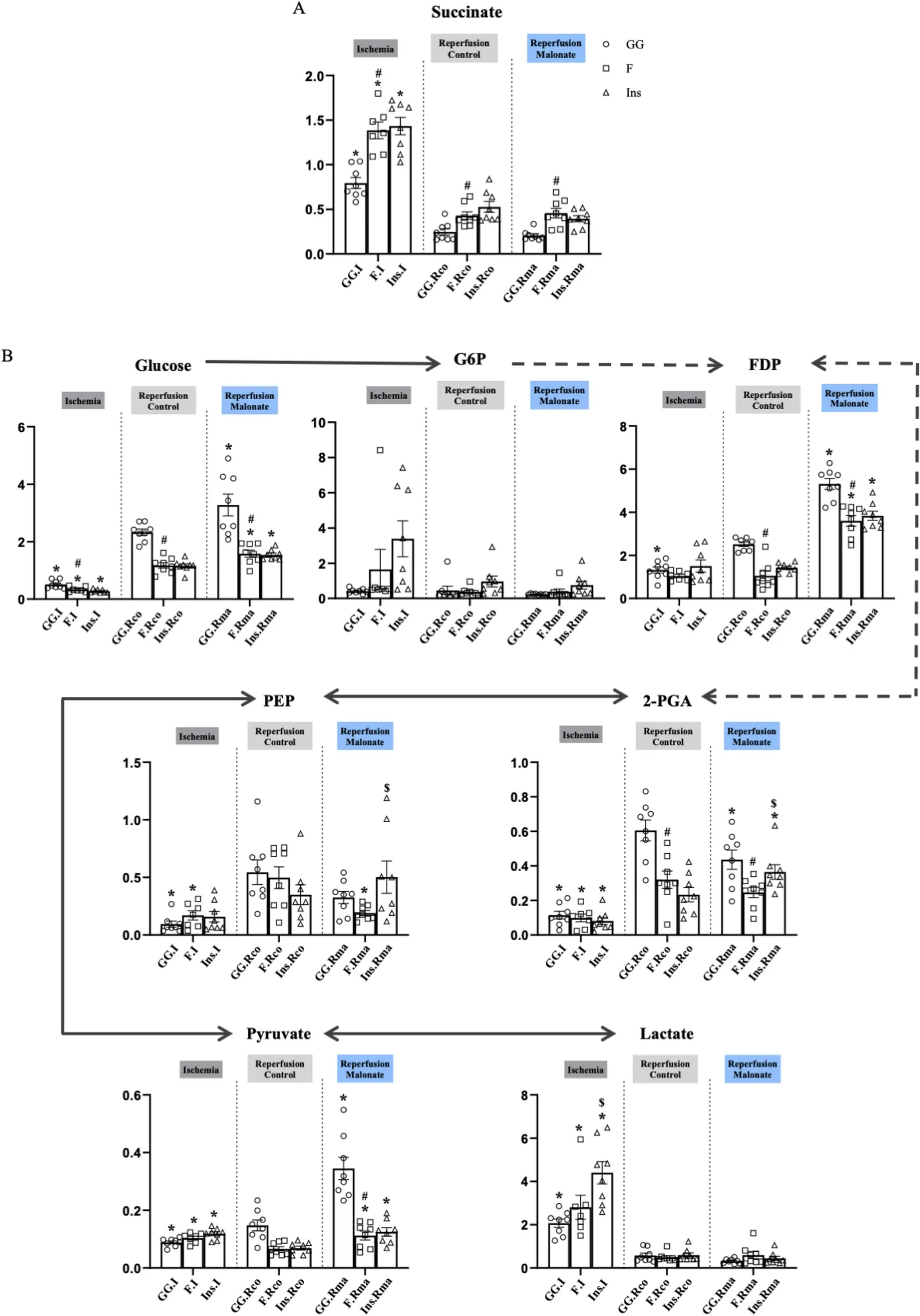
Malonate increased glycolytic intermediates and lactate in Ins group. Succinate and glycolytic intermediate levels were measured at the end of 30 min ischemia and 7 min after reperfusion with or without malonate. **A** Succinate levels in each group;** B** The levels of glucose, glucose 6-phosphate (G6P), fructose-1/2,6-bisphosphate (FBP), 2-phosphoglyceric acid (2-PGA), phosphoenolpyruvate (PEP), pyruvate, and lactate. *N* = 7 hearts for F.Ins group, *n* = 8 hearts for all other groups. A one-way ANOVA test followed by a Bonferroni post hoc analysis was conducted to identify differences between the reperfusion control (Rco) and ischemia (I) groups, as well as between Rco and reperfusion malonate (Rma) group, across all metabolic conditions. Additionally, comparisons were made between GG and F, as well as Ins and F, across all perfusion protocols by one-way ANOVA with a Bonferroni post hoc

Because the oxidation of accumulated succinate during ischemia is an important driver of RET-induced ROS during reperfusion, which oxidation is inhibited by malonate, we first focused on end-ischemic succinate levels trying to explain the loss of malonate protection with insulin. Ischemic accumulation of succinate was increased with the addition of fatty acids, possibly explaining the increased IRI with fatty acids, but no further increase in succinate was observed with insulin (Fig. 5A). The decrease in succinate upon reperfusion without or with malonate was also not different between the F and Ins groups (Fig. 5A). Finally, in a separate cohort of isolated hearts, we determined succinate release at the start of reperfusion for the F and Ins groups, showing that again there was no difference in the amount of succinate released from the heart between these two metabolic groups (Suppl. Figure 2D). Thus, it is unlikely that differences in succinate handling can explain the loss of malonate protection with insulin.

### Malonate and metabolic intermediates of glycolysis and pentose phosphate pathway (PPP)

We then focused on potential differences in metabolic intermediates of the glycolytic pathway at end-ischemia and during reperfusion between the Ins group and the F and GG groups (Fig. 5B), because of the observed malonate-induced increases in fructose-1,6-diphosphate as activator of glycolysis (Fig. 4). At end-ischemia, increased accumulation of lactate, with a trend for increased G6P levels, set the Ins group apart from the GG and F groups (Fig. 5B), suggesting increased anaerobic glycolysis during ischemia in the Ins group as compared to the GG and F groups Examining glycolysis effects at reperfusion, malonate increased glycolytic intermediates PEP and 2-PGA in the Ins group, suggestive of glycolytic bottlenecking, whereas decreases in these intermediates with malonate were observed for the GG and F groups, suggestive of glycolysis activation for these metabolic groups (see Suppl. Figure 3A).

Examining malonate’s effects on PPP intermediates showed that malonate increased all three PPP intermediates in the Ins group, whereas such an increase with malonate was not observed for the other metabolic groups (Suppl. Figure 3A–D). Thus, it is possible that the loss of malonate protection in the Ins group is due to alterations in the PPP. In order to examine this outcome, malonate protection in the Ins group was assessed during PPP inhibition with a G6PD inhibitor. However, for all cardiac IRI outcome variables, malonate protection was not recovered with continuously PPP inhibition (Suppl. Figure 3D–F), suggesting that the insulin-induced loss of malonate cardioprotection is not due to changes in PPP activity.

Thus, it seems that differences in glycolysis during ischemia and/or during early reperfusion with malonate associate with efficacy of malonate protection. Therefore, in the next series of experiments, we tested the involvement of glycolysis during reperfusion or during ischemia in malonate protection.

### Protection by malonate in Ins group restored with absence of glucose prior ischemia

**Fig. 6:**
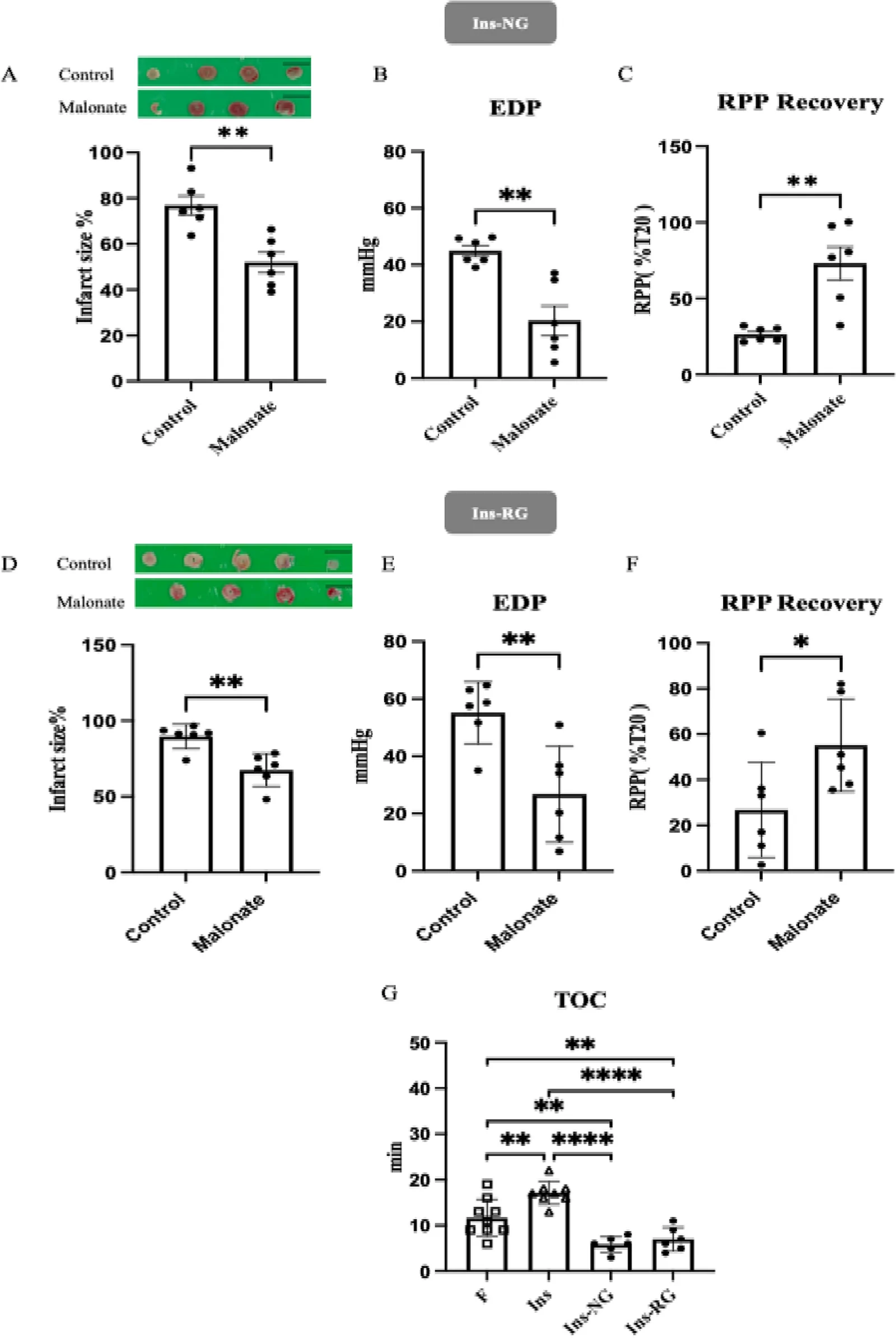
Malonate protection with insulin restored in absence of glucose before ischemia. Effects of malonate on cardiac IRI for hearts perfused with insulin, palmitate and glutamine but without glucose throughout the whole experiment (**Ins-NG**; **A-C** *n* = 6 hearts for each group) or only without glucose during baseline but with glucose present during reperfusion (**Ins-RG**; **D-F** *n* = 6 hearts for each group). **A, D** infarct size, scale bar, 1 cm; **B, E** end-diastolic pressure (EDP); **C, F** recovery of Rate Pressure Product (RPP). RPP calculated as (RPP_T140_—RPP_T20_)/RPP_T20_ × 100%. **G** Time of ischemic Contracture (TOC), defined as EDP > 11 mmHg, for F(*n* = 9), Ins(*n* = 8), Ins-NG (*n* = 6) and Ins-RG (*n* = 6) perfusions. T test was performed for panels A-C, E, and F, Mann–Whitney test was performed for panel D, a one-way ANOVA test followed by a Holm–Šídák post hoc analysis was conducted for panel G

To test whether changes in glycolysis can possibly explain the loss of malonate protection in the Ins group, malonate protection was examined in the absence of exogenous glucose throughout the whole experiment. Indeed, malonate was now able to protect again against cardiac IRI: IS was decreased by 32%, and recovery of cardiac function was improved (Fig. 6A–C). Additionally, malonate was now unable to increase the RISK pathway or mtHK, and was not associated anymore with increased 4-HNE levels in the Ins group (Suppl. Figure 4A–D).

The loss of malonate protection could be due to changes in glycolysis during reperfusion or during ischemia/baseline. To allow malonate effects on glycolysis during reperfusion still to occur, glucose was re-introduced at the onset of reperfusion only. However, also under those conditions, with glucose-only present during reperfusion, protection by malonate was reinstalled (Fig. 6D–F). Thus, it seems that protection by malonate is abolished in conditions when glucose and insulin are both present before ischemia in the Langendorff-perfused mouse heart.

Previous work has shown that with insulin and glucose present, there is increased glycogen loading of the heart that can distort cardioprotection, and thus maybe also malonate protection [38, 46]. We therefore determined time of contracture (TOC) during ischemia as an indirect measurement of cardiac glycogen [6, 7, 38, 41, 46] for the different metabolic conditions. Indeed, TOC was the largest for the condition where malonate lost protection (Fig. 6G). Additionally, cardiac glycogen levels for the F and Ins groups at start of ischemia, determined in a separate cohort of mice, demonstrated that indeed glycogen levels were approximately 3× higher in the hearts of the Ins group (Suppl. Figure 2E).

### Loss of malonate protection in insulin–glucose condition due to low pH at early reperfusion

**Fig. 7:**
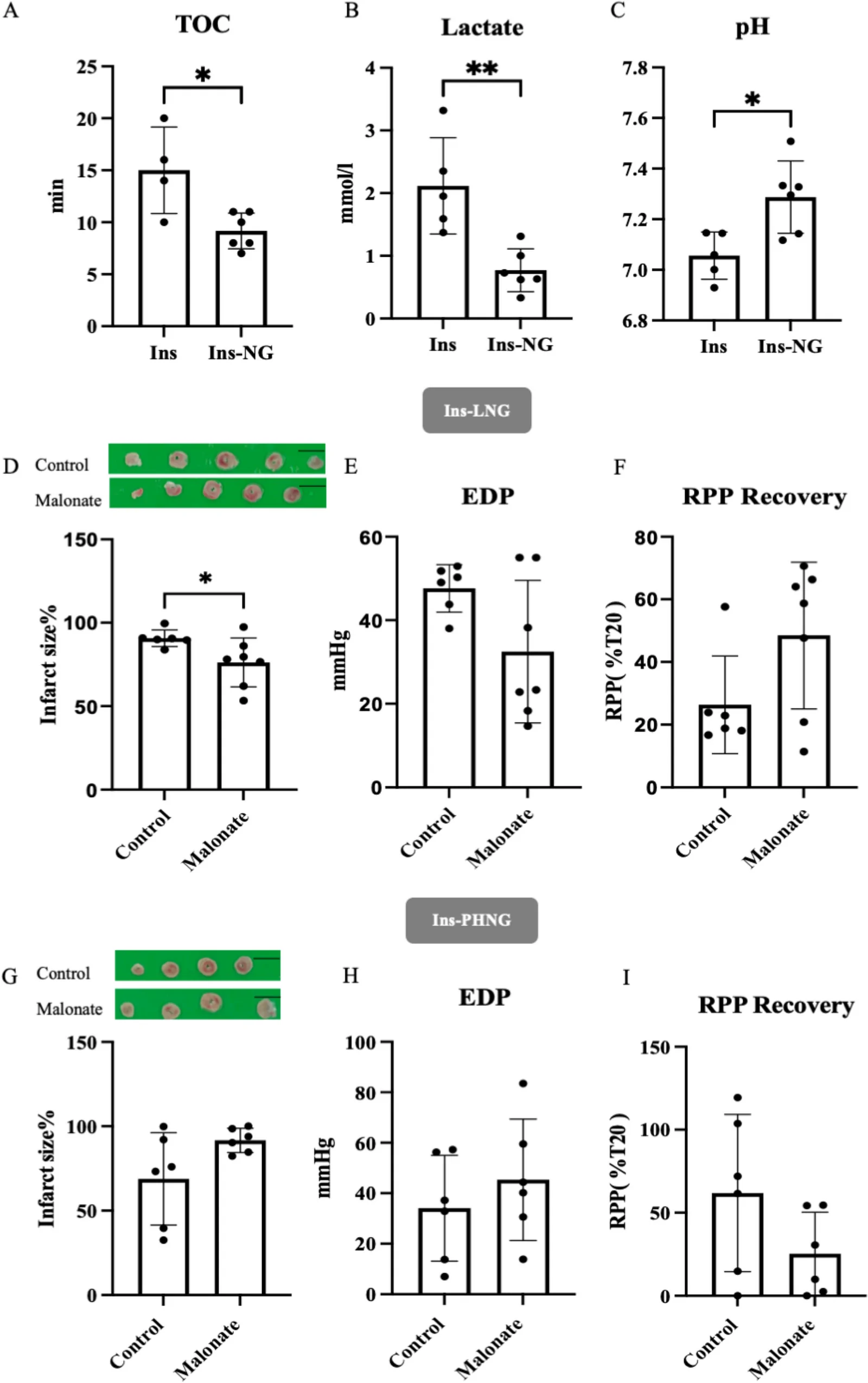
Glucose + insulin before ischemia caused low pH at reperfusion that abolished malonate protection. **A–C** Insulin + glucose (Ins) perfusion compared with Insulin without glucose (Ins-NG) perfusion for Time of Contracture during ischemia (**A** TOC, *n* = 4 hearts in Ins group, *n* = 6 hearts in Ins-NG group), and lactate (**B** *n* = 5 hearts for Ins group, *n* = 6 hearts for Ins-NG group) and pH (**C** *n* = 5 hearts for Ins group, *n* = 6 hearts for Ins-NG group) levels in venous effluent collected at start reperfusion. **D–F** malonate effect in presence of high lactate on infarct size (**D** scale bar, 1 cm), end-diastolic pressure (EDP; **E**) and recovery of Rate Pressure Product (RPP, **F**), *n* = 6 for control group, *n* = 7 for malonate group. Panels G through I represent the infarct size (scale bar, 1 cm), EDP and RPP in the group with glucose reperfusion (Ins-RG), *n* = 6 for each group. TOC, time where end-diastolic pressure rose above 11 mmHg during ischemia. RPP calculated as (RPP_T140_—RPP_T20_)/RPPT_20_ × 100%. T test was performed for panels A–I

Increased glycogen breakdown during ischemia should result in elevated lactate at end-ischemia [38], and facilitate enhanced anaerobic glycolysis during ischemia, resulting in increased proton loading of the heart at end-ischemia [7]. Elevations in lactate and proton at early reperfusion are therefore both likely candidates for distorting malonate protection. Therefore, we first determined whether adding glucose during baseline to Ins perfusate would indeed delay the time of ischemic contracture and increase lactate and proton release upon the onset of reperfusion. Indeed, adding glucose at baseline to Ins perfusate delayed ischemic contracture and increased lactate and lowered pH of venous effluent collected immediately upon start of reperfusion (Fig. 7A-C). We then tested whether high lactate at end-ischemia/early reperfusion abolished protection by malonate. However, malonate protection for the major IRI outcome variables was still observed (Fig. 7D-F). Finally, malonate protection was studied under conditions of low pH at the onset of reperfusion. Hearts were perfused with the Ins perfusate without glucose, and pH of the perfusate was lowered to 6.3 for the first 5 min of reperfusion. Malonate was now unable to protect the heart from IRI as shown for all three IRI outcome variables (Fig. 7G-I).

## Discussion

The major findings of the present study, in relation to the questions posed in the introduction, can be summarized as follows: 1) metabolic milieu is a major determinant of cardiac IRI, with infarct sizes ranging from 30% to over 60%, 2) malonate protection is critically dependent on the metabolic milieu of the heart, 3) malonate affects cardiac metabolomics at early reperfusion, increasing fructose-1,6-biphosphate, ATP, GTP, ribose and creatine phosphate, although these changes do not always correlate with protection by malonate, 4) malonate does affect cardioprotective signaling pathways, but malonate protection cannot be explained by changes in these signaling pathways, 5) presence of insulin plus glucose before the ischemic insult abolished malonate protection, probably because of glycogen loading of the heart before ischemia, with increased glycogen breakdown during ischemia driving acidosis at early reperfusion that then prevents malonate from being protective. Thus, our data suggest that severe acidosis at reperfusion may be a translational metabolic roadblock for malonate therapy in the clinical arena.

### Acidosis at onset reperfusion as mechanism blocking malonate protection

The loss of malonate protection with low pH at reperfusion could be explained by acidosis blocking 1) cellular uptake of malonate, 2) malonate cellular mechanisms of protection or 3) stimulating the release of succinate upon reperfusion such that little succinate remains to drive RET-ROS production and allow malonate to interfere. The metabolomics data clearly showed cellular uptake of malonate and effects of malonate independent of the metabolic condition, such as increasing e.g., FBP, acetylcarnitine (C2:0), citric acid, and ATP. Thus, these results argue against low pH blocking malonate cellular uptake. That acidosis would inhibit cellular malonate uptake is also unlikely considering the study by Prag et al. [29] showing acidosis to stimulate malonate uptake [29]. Although in that study [29], it was shown that acidified malonate improved protection against IR injury in glucose-only perfused isolated mouse hearts, protection by malonate was not tested for hearts perfused with insulin + glucose or for acidified perfusate, as is reported now in the current study. Acidosis could potentially block malonate protection when the final end-effector of malonate protection is the opening of the mitochondrial permeability transition pore (mPTP) [39] and acidosis can block such an opening. However, although it is commonly believed that acidosis prevents mPTP opening of mitochondria, this mainly applies to deenergized mitochondria [22]. In energized mitochondria, acidosis accelerates mPTP opening and can therefore possibly also worsen cardiac IRI. We also observed increased IRI with low pH at reperfusion, arguing against acidosis being protective during early reperfusion in our model. Alternatively, it is possible that in conditions of acidosis-induced accelerated mPTP opening, the protective action of malonate is lost because its ROS-lowering action is too much delayed relative to mPTP opening. This is likely when ROS is not an initial trigger for mPTP opening as has been noted in some studies [2, 3]. In these studies, the detachment of HK from mitochondria was suggested as trigger for mPTP opening. In the current study, an increased mtHK was indeed observed with malonate treatment. However, an increase in mHK with malonate also occurred in the Ins group, making it unlikely that changes in mtHK can explain the loss of malonate protection with insulin and glucose. In addition, the metabolomics data (Fig. 4) do not suggest that with the Ins perfusate, mitochondria are more energized because the levels of NADH, ATP, or creatine phosphate were not affected by the Ins perfusate. Finally, succinate release during the first min of reperfusion showed no difference between the F and Ins groups, arguing against increased succinate release in this metabolic condition as an explanation for the loss of malonate protection. In conclusion, acidosis at early reperfusion in the presence of insulin and glucose causes a loss of malonate protection that cannot be explained by impaired malonate uptake or increased succinate release but is probably related with acidosis worsening I/R injury. Subsequent research will be needed to further unravel the molecular mechanism causing the loss of malonate protection during acidosis.

### Malonate at reperfusion increases glycolysis and cardiac energetics for all metabolic conditions

This is the first report showing that malonate increased FBP for all metabolic conditions, raising the possibility that malonate protection against IRI is partly caused by increased FBP and consequently increased glycolysis [8]. FBP was recently reported to protect against cell death induced by potassium efflux during metabolic inhibition through increasing ATP, reducing AMPK activity and possibly ROS [48]. In the current study, we also observed increased ATP with decreased AMPK signaling with malonate-induced FBP increases under both F and Ins metabolic conditions. However, because these changes were still observed under the Ins condition, it is unlikely that these changes in ATP and AMPK can explain the protective effects of malonate.

Malonate-increased glycolysis was supported by the observation that malonate decreased the glycolytic intermediates of the lower part of glycolysis (PEP and 2-PGA) during reperfusion for the GG and F groups. However, the opposite was observed in malonate effects in the Ins group: malonate increased PEP and 2-PGA, indicative of a glycolysis pathway bottlenecking. In addition, intermediates of PPP only increased with malonate in the presence of glucose and insulin, which also may be explained by impairments in activation of the lower part of glycolysis, such that glycolytic intermediates of the upper part of glycolysis are shunted into the PPP. Thus, under the Ins condition, glycolysis seems not to be activated anymore by malonate, likely because it is already highly activated due to the presence of insulin. This poses the thesis that glycolysis activation could constitute malonate protection, also knowing that activated glycolysis is one of the major metabolic pathways underlying various cardioprotective interventions [53]. However, the experiments showing restoration of malonate protection under the Ins condition when glucose is not present, such that glycolysis cannot be activated, demonstrate malonate protection not to be mediated through glycolysis activation. Also, the maintenance of malonate efficacy when glucose is re-introduced at reperfusion in the Ins perfusate, a condition where glycolysis activation is maximal again, goes against glycolysis activation as part of the underlying mechanism of malonate protection. To summarize, although malonate seems to activate glycolysis, such activation is unlikely to be an underlying mechanism of the protection invoked by malonate administration.

### Valley of death for cardioprotective interventions: clinical efficacy

The field of cardioprotection has been struggling for over 35 years to bring a preclinically proven protective intervention successfully into the clinical arena. The mismatch between the experimental conditions of the preclinical model and the conditions prevailing in the clinical arena underlies this valley of death of translation [10, 17, 42]. In general, the translation to the clinic was executed too quickly without thorough testing of the observed protection in the preclinical model of cardiac IRI for different experimental conditions that bear relevance to the human condition during cardiac IRI. Therefore, before embarking on clinical testing of novel cardioprotective interventions, extended testing under different experimental conditions of its protective efficacy is now strongly recommended to prevent future failures.

Here we demonstrated that the novel protectant malonate, although protective under various metabolic conditions, lost protection in conditions of low pH during early reperfusion in the isolated mouse heart, probably caused by high glycogen breakdown during the ischemic period. This raises the question of whether this condition could also happen under in vivo and in humans. In a previous study, we demonstrated that the amount of glycogen present in the isolated mouse heart when perfused with insulin and glucose resembled the glycogen amount observed in the in vivo mouse heart [38]. Thus, the insulin + glucose glucose-perfused hearts mimic the in vivo condition of the mouse heart concerning cardiac glycogen content, thus bearing relevance for the in vivo condition. Diabetes, fasting, aging, and chronic hibernation are all known to increase cardiac glycogen content [4, 25–27] and may therefore create conditions under which malonate protection can be lost. Indeed, it was reported that malonate protection can fail under diabetic conditions for animal and human hearts [20, 37]. Therefore, it is advisable to perform further evaluation of malonate protection in these specific conditions, potentially needing higher doses of malonate, before embarking on clinical studies.

### Limitations

We used 10 mM lactate concentration in the perfusate trying to mimic the high lactate levels at start reperfusion in our hearts. This concentration of lactate was an estimate based on previous cardiac lactate measurements in (insulin + glucose) perfused mouse hearts at end-ischemia of 0.9 μmol/mg dried heart weight (See Fig. 4 of [36]). Assuming that most of this lactate is released during the first min of reperfusion with an averaged flow of 2.5 ml/min and the averaged dried weight of the mouse heart being 30 mg, this translates into 27 μmol lactate in 2.5 ml, corresponding to 10.8 mM lactate concentration in the total fluid compartment of the heart.

Although malonate protection was observed for three out of the four metabolic conditions, indirectly showing that malonate reduced SDH oxidation of succinate during reperfusion assuming that RET-ROS production by succinate oxidation drives IRI, direct proof of inhibition of succinate oxidation by malonate during early reperfusion is lacking in the current study. Future experiments should determine cardiac and effluent succinate levels at reperfusion times earlier than 7 min to directly test malonate-induced reduction in succinate oxidation for the separate metabolic conditions of the Langendorff-perfused mouse hearts.

Hearts were perfused for the first 5 min of reperfusion with a low pH (6.3) to study whether the low pH could explain the loss of malonate protection. The lower pH per se was without effect on infarct size (*76* ± *10% (INS-NG group) versus 69* ± *27% (INS-PHNG group), p* = *0.52).* Although a lower pH is commonly protective in isolated cell studies, the effects of low pH reperfusion on IRI in intact hearts are much less clear. Protection in intact heart is mostly observed for ≤ 3 min low pH perfusion, and disappeared, or even becomes damaging, with longer low pH reperfusion times [19]. Further experiments will be needed to examine whether the loss of protection by low pH reperfusion observed in the current study can be explained by the employed timing of the low pH reperfusion intervention.

## Conclusion

Malonate is a novel metabolic therapy against cardiac and brain IRI with promise toward clinical application for the treatment of acute cardiac infarct and stroke patients. Acknowledging the necessity of extensive preclinical testing of malonate efficacy under different conditions relevant to the clinical scenario before embarking on clinical application, in the present study, various metabolic conditions of the heart were examined. While malonate offers robust protection against cardiac IRI across various metabolic environments, its efficacy is compromised under conditions of high ischemic glycogen breakdown, which leads to severe acidosis at the onset of reperfusion that abolished malonate protection. Knowing that diabetes, aging, hibernation, and fasting can all raise cardiac glycogen content, these specific conditions warrant preclinical testing before applying malonate therapy in the older, diabetic, hibernated, and/or fasted patient.

## Supplementary Information

Below is the link to the electronic supplementary material.

Supplementary file1 (DOCX 29469 KB)

## Data Availability

The data underlying this article will be shared on reasonable requests to the corresponding author.
