# Supplementary material for: Metabolic dependency of malonate protection against cardiac ischemia–reperfusion injury
Source: Basic Res Cardiol. 2026 Aug 17;121(5):863–82. doi: 10.1007/s00395-026-01206-4 (PMC13593634; doi:10.1007/s00395-026-01206-4)
Supplement: Supplementary file 1 [file 395_2026_1206_MOESM1_ESM.docx]

**Supplementary Files**

**Supplementary Table 1** The baseline cardiac characteristics of Langendorf-perfused mouse hearts at T0 for each metabolic group of series 1, shown in Figure 1

|  | Flow (ml/min/G_ww_) | |  | P_per_ (mmHg) | |  | EDP  (mmHg) | |  | DLVP  (mmHg) | |  | HR  (bpm) | |  | RPP  (mmHg/min) | |
| --- | --- | --- | --- | --- | --- | --- | --- | --- | --- | --- | --- | --- | --- | --- | --- | --- | --- |
|  | Mean | SD |  | Mean | SD |  | Mean | SD |  | Mean | SD |  | Mean | SD |  | Mean | SD |
| ***G*** |  |  |  |  |  |  |  |  |  |  |  |  |  |  |  |  |  |
| Control | 18.3 | 2.2 |  | 81 | 4 |  | 4.2 | 1.4 |  | 112 | 15 |  | 385 | 26 |  | 45616 | 5741 |
| Malonate | 15.7 | 3.4 |  | 81 | 4 |  | 3.7 | 1.5 |  | 105 | 17 |  | 376 | 28 |  | 42090 | 8050 |
| ***GG*** |  |  |  |  |  |  |  |  |  |  |  |  |  |  |  |  |  |
| Control | 17.0 | 2.8 |  | 78 | 3 |  | 4.6 | 1.6 |  | 110 | 14 |  | 376 | 45 |  | 41249 | 8070 |
| Malonate | 17.7 | 1.9 |  | 82 | 3 |  | 4.6 | 0.6 |  | 112 | 10 |  | 399 | 43 |  | 44557 | 4156 |
| ***F*** |  |  |  |  |  |  |  |  |  |  |  |  |  |  |  |  |  |
| Control | 14.4** | 5.4 |  | 83 | 7 |  | 3.2 | 1.7 |  | 109 | 6 |  | 351 | 52 |  | 38031 | 5377 |
| Malonate | 13.0 | 1.6 |  | 82 | 1 |  | 4.6 | 1.9 |  | 106 | 12 |  | 320 | 24 |  | 33630 | 3105 |
| ***Ins*** |  |  |  |  |  |  |  |  |  |  |  |  |  |  |  |  |  |
| Control | 13.4** | 3.7 |  | 81 | 4 |  | 3.6 | 0.8 |  | 104 | 16 |  | 339* | 22 |  | 35465 | 6871 |
| Malonate | 13.2 | 1.8 |  | 80 | 3 |  | 3.8 | 1.3 |  | 117 | 17 |  | 347 | 24 |  | 40444 | 6241 |

Flow (ml/min/G_ww_), coronary flow measured at the onset of baseline; Pper, perfusion pressure; EDP, end diastolic pressure; DLVP, developed left ventricular pressure; HR, heart rate, RPP, rate pressure product (DLVP × HR). N=8-19 hearts per group. Kruskal-Wallis test with a Dunn’s post hoc (Flow, EDP, DLVP and RPP) or one-way ANOVA with Holm-Šídák post hoc test (P_per_ and HR) was performed for detecting differences between groups. Statistical significance: *P < 0.05, **P < 0.01 vs control in the G group.

**Suppl Fig 1.** **Malonate effect on stress signalling pathways and oxidative stress in hearts perfused with F and Ins perfusates.** **(A)** RISK pathway (Akt, GSK3β, mTOR) activation at early reperfusion with or without malonate for the F and Ins metabolic condition. **(B)** AMPK activation at early reperfusion with or without malonate for F and Ins condition. **(C, D)** Mitochondrially-bound hexokinase 1 and 2 at early reperfusion with or without malonate for F and Ins group. **(E)** oxidative stress marker 4-HNE at early reperfusion and end-ischemia for F and Ins group. **(F)** 4-HNE at early reperfusion with or without malonate for F and Ins grou. **(G)** western blots of the targets depicted in A-F. **(H)** western blot showing 4-HNE at reperfusion and end-ischemia. ***F.Rco*** or ***F.Rma***, hearts perfused with F perfusate without and with malonate, respectively. ***Ins.Rco*** or ***Ins.Rma***, hearts perfused with Ins perfusate without and with malonate, respectively. N=6 hearts for all groups. one-way ANOVA with Holm-Šídák post hoc test (A-D and F) or Kruskal-Wallis test with a Dunn’s post hoc (E) was performed for detecting differences between groups. Statistical significance: *P < 0.05, **P < 0.01, ***P < 0.001.

**Suppl Fig 2. (A)** Plasma insulin levels in in vivo rats. **(B)** The effects of malonate were evaluated in vivo using a rat left anterior descending coronary artery (LAD) ligation model. Rats underwent 25 min of regional ischemia followed by 120 min of reperfusion. Malonate (160 mg/kg) was infused intravenously (0.3 mL/kg/min) starting the last 5 min of ischemia, ending at 5 min of reperfusion. Myocardial injury was assessed by determining infarct size (IS) and the area at risk (AAR). AAR as a percentage of the left ventricle (LV). IS as a percentage of the AAR. **(C)** Malonic acid levels in cardiac tissue at 7 min reperfusion for the GG, F and Ins metabolic groups treated without (Rco) or with 5 mM malonate (Rma) during reperfusion. Malonic acid was normalised to the internal standard of D4-citrate and dried tissue weight. **(D)** Effluent succinate levels of hearts during the first minute of reperfusion for hearts perfused with F and Ins perfusates. **(E)** Cardiac glycogen levels in hearts perfused with F and Ins perfusates at the end of the 20-min baseline period. One-way ANOVA followed by Holm-Sidak’s post hoc test (C) was performed for detecting differences between groups. T-test (A, B, D and E) was performed to compare the differences between two groups. Statistical significance: *P < 0.05, **P < 0.01, ****P < 0.001, ****P < 0.0001 vs Control group.

**Suppl Fig 3. Pentose phosphate pathway (PPP) and malonate treatment effects.** **(A)** Schematic diagram of the proposed effects of malonate on glycolysis and PPP during early reperfusion in hearts without or with insulin. **(B-D)** PPP intermediates at end-ischemia and at 7 min reperfusion without and with malonate treatment for the GG, F and Ins metabolic condition. Panel B, C and D represent the levels of gluconate-6P, ribose-5P and sedoheptulose-7p, respectively. N=7 hearts for F.Ins group, n=8 hearts for all other groups. A one-way ANOVA test followed by a Bonferroni post hoc analysis was conducted to identify differences between the reperfusion control (Rco) and ischemia (I) groups, as well as between Rco and reperfusion malonate (Rma) group, across all metabolic conditions. Additionally, comparisons were made between GG and F, as well as Ins and F, across all perfusion protocols by one-way ANOVA with a Bonferroni post hoc. **(E-G)** malonate effects with Ins perfusate in the presence of the PPP-inhibitor G6PDi-1 on cardiac IRI outcome variables of infarct size (E; scale bar, 1 cm), end-diastolic pressure (F, EDP) and the recovery of Rate-Pressure-Product (G, RPP). N=6 hearts for each group. RPP calculated as (RPP T140 – RPP T20)/RPPT20 × 100%. T test was conducted for panels E-G. Statistical significance: *P < 0.05 vs reperfusion control group in all metabolic conditions; $P < 0.05, Ins vs F in all perfusion protocols, with or without malonate treatment; #P < 0.05, F vs GG in all perfusion protocols, with or without malonate treatment.

**Suppl Fig 4. Malonate effect on stress signalling pathways and oxidative stress at early reperfusion in hearts perfused with palmitate, glutamine and insulin but without glucose throughout the whole experiment (Ins-NG).** After 30 min ischemia and 7 min reperfusion, heart tissue was collected, homogenized and western blotted. **(A)** Malonate effect on RISK pathway (AKT, GSK3β and mTOR) at early reperfusion. **(B)** Malonate effect on mitochondrial hexokinase 1 (mtHK1) and mitochondrial hexokinase 2 (mtHK2) at early reperfusion. **(C)** Malonate effect on the level of 4-HNE as indicator of oxidative stress. **(D)** Western blot images for panel A, B and C. N=6 hearts for each group. T test was performed for panels A and B (mtHK2), Mann-Whitney test was performed for panel B (mtHK1) and C.
